# Characterization of a New Variant in *ARHGAP31* Probably Involved in Adams–Oliver Syndrome in a Family with a Variable Phenotypic Spectrum

**DOI:** 10.3390/genes15050536

**Published:** 2024-04-24

**Authors:** Carlo Santaniello, Alice Faversani, Luigi Corsaro, Giulia Melloni, Silvia Motta, Elena Mandorino, Davide Sacco, Sabine Stioui, Fulvio Ferrara, Davide Barteselli, Dario De Vita, Debora Manuelli, Lucy Costantino

**Affiliations:** 1 Laboratory of Medical Genetics, Centro Diagnostico Italiano, 20147 Milan, Italy; carlo.santaniello@cdi.it (C.S.); alice.faversani@cdi.it (A.F.); luigi.corsaro@ext.cdi.it (L.C.); giulia.melloni@cdi.it (G.M.); silvia.motta@cdi.it (S.M.); elena.mandorino@cdi.it (E.M.); davide.sacco@cdi.it (D.S.); sabine.stioui@cdi.it (S.S.); barteselli.davide@gmail.com (D.B.); dario.devita@cdi.it (D.D.V.); debora.manuelli@cdi.it (D.M.); 2 Department of Brain and Behavioral Science, Università Degli Studi di Pavia, 27100 Pavia, Italy; 3 Integrated Laboratory Medicine Services, Centro Diagnostico Italiano, 20147 Milan, Italy; fulvio.ferrara@cdi.it

**Keywords:** Adams–Oliver syndrome, *ARHGAP31*, clinical exome sequencing, limb defects

## Abstract

Adams–Oliver syndrome is a rare inherited condition characterized by scalp defects and limb abnormalities. It is caused by variants in different genes such as *ARHGAP31*. Here, we used an interdisciplinary approach to study a family with lower limb anomalies. We identified a novel variant in the *ARHGAP31* gene that is predicted to result in a truncated protein with a constitutively activated catalytic site due to the loss of 688 amino acids involved in the C-terminal domain, essential for protein auto-inhibition. Pathogenic variants in *ARHGAP31* exon 12, leading to a premature protein termination, are associated with Adams–Oliver syndrome. Bioinformatic analysis was useful to elucidate the impact of the identified genetic variant on protein structure. To better understand the impact of the identified variant, 3D protein models were predicted for the ARHGAP31 wild type, the newly discovered variant, and other pathogenetic alterations already reported. Our study identified a novel variant probably involved in Adams–Oliver syndrome and increased the evidence on the phenotypic variability in patients affected by this syndrome, underlining the importance of translational research, including experimental and bioinformatics analyses. This strategy represents a successful model to investigate molecular mechanisms involved in syndrome occurrence.

## 1. Introduction

Adams–Oliver syndrome (AOS [MIM 100300]) is a rare inherited multiple malformation syndrome with an estimated frequency of 1 individual out of 225,000 live-born infants [1]. The main features of AOS are two congenital defects: aplasia cutis congenita (ACC), with terminal transverse limb defects (TTLDs). ACC is mostly present on the scalp, and some patients show bone hypoplasia. The spectrum of TTLDs includes syndactyly (osseous and/or limited to the skin), split hand or foot, and polydactyly. In addition, some patients show small or absent nails. This syndrome may be associated with other abnormalities, such as congenital heart defects, ocular anomalies, or defects involving the central nervous system, as recently reported [2]. AOS is genetically heterogeneous, and several genes have been identified as causing the condition. Sporadic AOS cases and autosomal dominant (AD) forms are associated with variants in the *ARHGAP31*, *RBPJ*, *NOTCH1*, and *DLL4* genes, whereas variants in the *DOCK6* and *EOGT* genes are correlated with AOS autosomal recessive (AR) forms. Four causal genes (*NOTCH1*, *RBPJ*, *EOGT*, and *DLL4*) are involved in NOTCH signaling, and the NOTCH pathway plays a key role in AOS pathogenesis and development [3]. The *DLL4* gene encodes for a specific protein–ligand of the NOTCH receptors (NOTCH 1-4), while RBPJ is the principal transcriptional regulator for NOTCH signaling, which modulates its transcriptional complex [4]. *EOGT* encodes for an enzyme that transfers N-acetylglucosamine to serine or threonine residues to proteins containing eukaryotic (EGF)-like domains, such as NOTCH receptors [5]. Meanwhile, *DOCK6* and *ARHGAP31* are not directly related to the NOTCH pathway; rather, they encode for regulatory proteins that control the activity of the Rho GTPases Rac1 and Cdc42, involved in the maintenance of the actin cytoskeleton [6]. ARHGAP31 belongs to the Rho GAPs (GTPase-activating proteins) family, which controls the activation of GTPase proteins whose cycle switches between an inactive form (bound GDP) and an active one (bound GTP). ARHGAP31 regulates two GTPases, Rac1 and Cdc42, involved in protein trafficking and cell growth [6]. Rac1 and Cdc24 play an important role in cell diffusion, polarized lamellipodia formation, and cell migration [6]. Currently, four pathogenic *ARHGAP31* variants have been reported in families with Adams–Oliver syndrome type 1 cases: c.2047C>T, c.2063_2064insTT, c.3260del, and c.2182C>T [3,6,7]. All these variants are in exon 12 of the *ARHGAP31* gene and introduce a premature stop codon that causes the synthesis of an altered protein. In particular, the protein loses most of the amino acids encoded by exon 12, which constitutes the C-terminal domain, essential for the auto-inhibition mechanism. Truncating variants cause a significant inactivation of Cdc42 and alter Cdc42 and/or Rac1 [6].

Next-generation sequencing (NGS) approaches, such as clinical exome sequencing (CES), are demonstrating increasing usefulness in investigating rare diseases with complex and/or unknown genetic etiologies, and/or confirming clinical suspects. The identification of mechanisms and pathways altered by new variants involved in rare diseases is essential to define their effects. Experimental and bioinformatic approaches should be used to address this issue.

In this study, we used a genome-wide approach to investigate the case of a 33-year-old woman with lower extremity digit hypoplasia of both feet. Family analysis showed the presence of a heterozygous variant in *ARHGAP31* and highlighted the variability in clinical features among AOS patients.

## 2. Materials and Methods

All experiments were carried out according to the manufacturer’s instructions and laboratory best practices.

### 2.1. Patients

The proband, her parents, and her brother received genetic counseling at the Department of Molecular Genetics and Cytogenetics of the Centro Diagnostico Italiano (CDI) in Milan (IT). Informed consent was obtained from all family members, and two blood samples were taken from all of them. Six control subjects were recruited for gene expression analysis. Informed consent was obtained from all of them.

### 2.2. DNA Purification

Genomic DNA (gDNA) was purified from peripheral blood lymphocytes (PBLs) using the MagCore^®^ Genomic DNA Whole Blood Kit (RBC Bioscience, Birmingham, UK). The quality of the isolated DNA was evaluated by a NanoDrop One spectrophotometer (Thermo Fisher Scientific, Waltham, MA, USA).

### 2.3. Clinical Exome Sequencing (CES)

Clinical exome analysis was performed using the Clinical Exome Solution v2 kit (SOPHiA GENETICS, Lausanne, CH, Switzerland), which investigates the coding sequences of 4490 OMIM genes and their flanking intron regions (±5 nucleotide bases). The libraries were sequenced by 251 paired-end reads on the Illumina MiSeq DX platform (Illumina, San Diego, CA, USA). De-multiplexed FASTQ files, which originated from the sequencing, were aligned with the reference GRCh37 genome, and variant calling was performed by the SOPHiA DDM^TM^ platform (SOPHiA GENETICS, Lausanne, CH, Switzerland). This method did not allow the detection of copy number variations (CNVs) in target regions. Accuracy: 99.99%. Sensitivity: 99.45%. Specificity: 99.99%. Average % of the target region with a depth of > 50x: > 96%. The interpretation of clinical relevance variants was performed following the ACMG and ACGS guidelines [8,9]. The nomenclature was drawn up using HGVS v21.0.2.

### 2.4. Sanger Sequencing of Specific Region

The variants of clinical interest were confirmed using gDNA derived from a second blood sample. Primers (*ARHGAP31*-ex12_Fw, 5′ GGCCCTTTATTCCCTCAGA 3′, *ARHGAP31*-ex12_Rv, and 5′ TGAAAGCAGGACTGGAGTTG 3′) were designed by Primer3Plus–Bioinformatics (Appendix A), and the FastStart Taq DNA polymerase kit (Hoffmann-La Roche, Basel, CH, Switzerland) was used for the amplification of the region of interest (annealing temperature: 59 °C). The PCR product was evaluated using 2% gel electrophoresis by the E-Gel Power Snap Electrophoresis System (Thermo Fisher Scientific, Waltham, MA, USA). Sanger sequencing was performed by BigDye™ Terminator v3.1 chemistry on the Sanger sequencing platform 3500 Genetic Analyzer (Thermo Fisher Scientific, Waltham, MA, USA). The sequences were analyzed by Sequencing Analysis Software v.6 (Thermo Fisher Scientific, Waltham, MA, USA).

### 2.5. Array-Based Comparative Genomic Hybridization (aCGH) Profiling

Analysis of the CNVs was performed via a whole-genome platform, and the CNV data were generated using the Agilent GenetiSure Dx Postnatal CGH+SNP 180K platform (Agilent Technologies, Santa Clara, CA, USA), which has a 4 × 180 K resolution (mean resolution: 20 kb). Commercial human genomic DNA (human reference DNA, male and female; Agilent Technologies) was used as a normal reference. The analyses of the data were performed using Agilent CytoDx 1.2.0.9 (Agilent Technologies) with the detection algorithm ADM-2 (Aberration Detection Method 2). A change in the number of copies is defined by a shift from the normal value of at least 5 consecutive probes, while loss of heterozygosity (LOH) is defined by a displacement of at least 100 SNP probes. The map locations refer to the Genome Reference Consortium Human Build 19 assembly (GRCh19). The classification of variants with clinical relevance was carried out following the ACMG 2020 standards and guidelines [10].

### 2.6. RNA Purification

Peripheral blood mononuclear cells (PBMCs) were isolated by stratification with Ficoll-Paque PLUS (Eppendorf, Hamburg, DE, Germany). RNA (>200 bases) was isolated from PBMC pellets using the QIAamp RNA Blood Mini Kit (Qiagen, Hilden, DE, Germany). The quality of the isolated RNA was evaluated by a NanoDrop One spectrophotometer (Thermo Fisher Scientific, Waltham, MA, USA).

### 2.7. Gene Expression Analysis

An amount of 0.5 µg of RNA was reverse-transcribed using the High-Capacity cDNA Reverse Transcription Kit (Thermo Fisher Scientific, Waltham, MA, USA) according to the manufacturer’s instructions. Gene expression analysis was performed by RT-qPCR using a TaqMan Fast Advanced Master Mix (Thermo Fisher Scientific, Waltham, USA), specific TaqMan probes (Thermo Fisher Scientific, Waltham, MA, USA; *ARHGAP31*, Hs00393361_m1, *Cdc42*, Hs00918044_g1, *Rac1*, Hs00251654_m1, *CCND1*, and Hs00765553_m1), and QuantStudio 5 real-time PCR (Thermo Fisher Scientific, Waltham, MA, USA). Gene expression levels were obtained by comparison with the expressions of three housekeeping genes (*B2M*, Hs00187842_m1; *ACTB*, Hs01060665_g1; and *GADPH*, Hs00266705_g1 (Thermo Fisher Scientific, Waltham, MA, USA)) and using GeNorm software v3 [11].

### 2.8. Bioinformatic Analysis

All six ARHGAP31 proteins, five with variants and the wild type, were computed using AlphaFold 2.0 [12], Appendix A. Elaborations were performed using the Colab [13] notebook published by DeepMind, which received the amino acid sequence determined by Mutalyzer 3 as input [14] and produced the PDB file (Appendix A) as output with information on the tertiary structures of the proteins.

AlphaFold monomer implementation was performed to derive the monomer structures using the following protein structure databases: BFD, MGnify, PDB70, PDB, UniRef30 (FKA UniClust30), and UniRef90, (Appendix A).

The relaxed best models were generated after the 5th iteration with the default configuration of AlphaFold. This configuration only provided the best model based on the pLDDT (predicted local distance difference test). The six tertiary structures were compared with each other, resulting in a total of 15 tests to check which structures were similar to one another and how much the structures created by the variants differed from the wild type. To check whether the derived proteins were differently dislocated in 3D space, a geometric comparison was applied, which is typically used to check the correspondence between solute proteins and the crystallographic structure [15].

The sequence-dependent main methods used were root-mean-square deviation (RMSD) comparison and the global distance test (GDT).

The first method suffers from dependence on the largest errors, and the second requires the definition of a distance threshold. The RMSD comparison was performed using the BioPython library Bio.PDB [16] with the calculated iterative weighted superimposition and the associated superposition error.

The GDT method was applied with a variable threshold from 1 to 20 ångström (Å), which was higher than the value of 3.8 Å of the corresponding atom in the experimental structure when comparing a solved protein with a derived one [15].

Both calculations were performed using the carbon α (Ca) position as a reference, assuming a strict one-to-one correspondence between the target and model residues.

The tertiary structures were then superimposed with pyMol* v2.6 [17] and Python v3.9 (Appendix A) and generated as a single image so that they were aligned in the image window. All charts and data manipulation were generated using Python v3.9 and R v3.4.

## 3. Results

### 3.1. Clinical Information 

**Figure 1:**
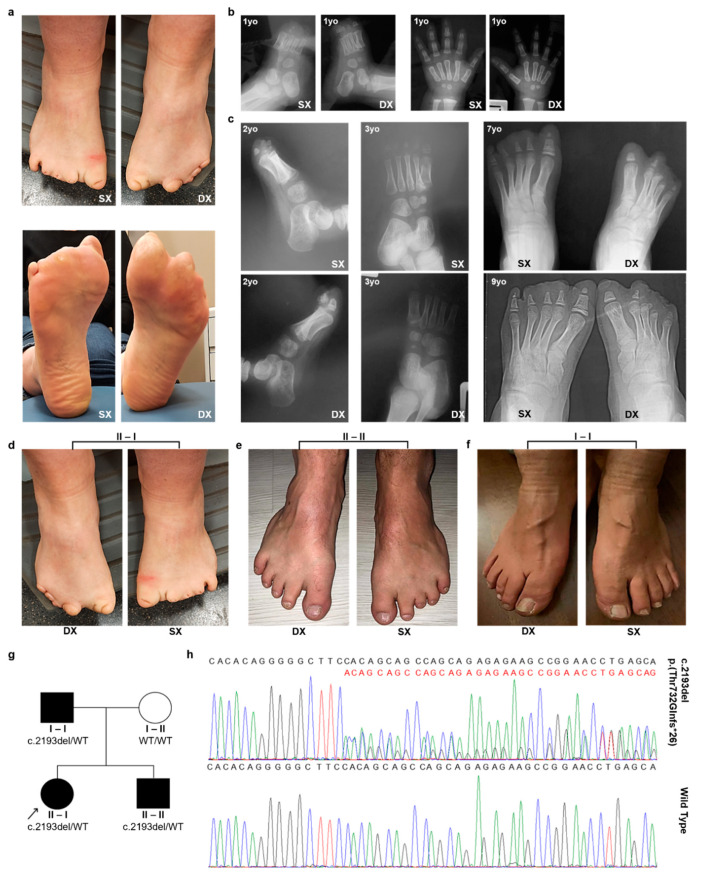
The proband and family members’ phenotypic and molecular features. (**a**) Images of the proband’s feet showing lower extremity finger hypoplasia. (**b**,**c**) The proband underwent various X-ray examinations. Panel (**b**) displays the proband’s hand and foot X-ray images at 1 year old. Her foot X-ray images at different ages (2, 3, 7, and 9 years old) are shown in panel (**c**). Panels (**d**–**f**) report the images showing the foot defects of the proband (II-I; **d**), her brother (II-II; **e**), and her father (I-I; **f**). Family pedigree is shown in panel (**g**). Individuals with foot anomalies, regardless of their variability, are shown in black (**g**). The family pedigree also shows the segregation of c.2193del p.(Thr732Glnfs*26). Variant carriers are reported as c.2193del/WT. The black arrow indicates the proband. Square: male; circle: female; black: affected individuals; blank: unaffected individuals. (**h**) DNA sequence electropherograms for WT and altered *ARHGAP31* are shown.

A 33-year-old Caucasian woman with lower limb anomalies (Figure 1a) asked the Laboratory of Medical Genetics of the Centro Diagnostico Italiano to assess her reproductive risk. She reported that she was born from an uneventful pregnancy. Her mother remembered that she took an antiemetic drug only once during pregnancy. At birth, her vital parameters were normal, while both her feet displayed lower extremity digit hypoplasia. No other clinical signs/symptoms were noted. She reported that she had undergone several analyses. She underwent foot X-ray analyses at different ages (Figure 1b,c). At the age of one year, an X-ray examination of both feet and hands revealed dysmorphic ossification nuclei in all the proximal phalanges of the left foot, rudimentary proximal phalanges of the right foot, and mild dimensional reductions in the second finger intermediate and ungual phalanges of the left hand (Figure 1b). At three years of age, she underwent surgical intervention: cutaneous z-plasty for small congenital foot grooves. At the age of 4, a borderline growth delay and recurrent airway infections were the reason for performing some additional tests, such as constitutional karyotype, a sweat chloride test, a thoracic X-ray, and a lymphocyte sub-population analysis. The constitutional karyotype results were normal in the 40 metaphases obtained from blood peripheral lymphocytes. The sweat chloride test, thoracic X-ray, and lymphocyte sub-population results were normal too. Blood tests for the suspicion of malabsorption were also performed, and the results were normal. At the age of 29, she underwent the last X-ray evaluation of both feet, showing dimensional reduction in all the proximal phalanges and the absence of all the distal ones. Moreover, prior to our evaluation, she already had an echocardiogram and a renal artery Doppler ultrasound evaluation because of borderline hypertension. The results were substantially normal, except for a mild tricuspid insufficiency, without signs of pulmonary hypertension.

In our clinical evaluation, she showed evident hypoplastic lower extremity fingers in both feet (Figure 1a,d). Both hands appeared normal, and no additional signs could be found. She reported that her family history was unremarkable. However, at the time of her blood collection, a detailed clinical examination was performed on her parents and her brother. Her brother was found to have a shortening of the toes of both feet, especially the third and fourth toes of the left foot (Figure 1e). Bilateral partial syndactyly of the second and third toes was noted both in the brother and the father (Figure 1e,f). Her father had partial second and third lower extremity digit syndactyly (Figure 1f). In a clinical re-examination, the proband revealed a very mild thinning of the cutis, without alopecia, at the palpation of the vertex region on her cranium. No cranial alterations were identified in either the brother or the father. The brother and father underwent echocardiograms, the results of which were normal. No additional clinical signs were found in either the parents or the brother. The proband’s parents were not blood relatives. Based on the family analysis, the phenotype had an apparent autosomal dominant inheritance pattern, even if with a variable severity of lower limb defects. The pedigree structure of the family is shown in Figure 1g. In Table 1, the family members’ personal and clinical information is reported.

### 3.2. Clinical Exome Analysis

To investigate the genetic causes involved in the family phenotype, we performed a clinical exome sequencing analysis using DNA derived from all the family members. This analysis identified the presence of a heterozygous variant in the *ARHGAP31* gene (NM_020754.4) in both siblings (II-I and II-II) and their father (I-I): NM_020754.4:c.2193del NP_065805.2:p.(Thr732Glnfs*26), (Figure 1g). The identified alteration is a frameshift variant (Figure 1h) located in exon 12 of *ARHGAP31,* which inserts a premature STOP codon after 26 codons. At the protein level, this variant predicted the formation of a truncated protein, with the loss of 688aa involved in the C-terminal domain. This variant was not reported in clinical databases, such as ClinVar and LOVD (Appendix A), and was absent in control populations (gnomAD, Appendix A). The presence of the variant was confirmed by Sanger sequencing. The proband’s mother did not show this alteration, confirming that the siblings inherited it from their father. Different variants in *ARHGAP31* exon 12, leading to premature termination of the translated protein, have been classified as pathogenic [6] and associated with an autosomal dominant form of AOS.

### 3.3. Protein Inference

**Figure 2:**
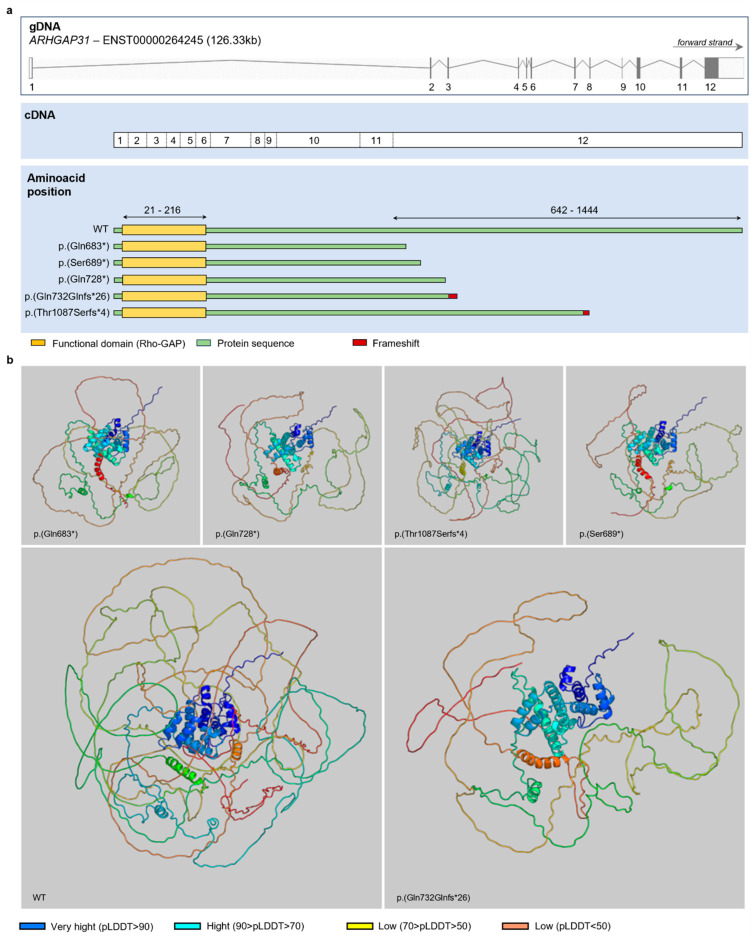
ARHGAP31 variant protein structures. (**a**) Schematic representation of *ARHGAP31* cDNA and protein structures. In particular, the Figure shows the protein structure of ARHGAP31 wild type and that predicted for ARHGAP31 variants. (**b**) Protein tertiary structures resulting from the ARHGAP31 variants and the wild type. Color coding reflects the pLDDT, where very high values in blue are representative of high confidence, while lower values in orange indicate very low confidence.

To understand the protein effect of the newly identified ARHGAP31 variant, we compared its protein structure with the wild-type one and that of four other ARHGAP31 variants involved in AOS (Figure 2a) by protein inference. The putative protein’s tertiary structure resulting from the ARHGAP31 variants as well as the wild-type protein tertiary structure, identified with Q2M1Z3 by Uniprot [18], were inferred using AlphaFold and are illustrated in Figure 2b. Each derived structure was superimposed onto the wild type, and all were aligned in the same orientation. The coloration of protein segments corresponds to the pLDDT test, a metric indicating the predicted local distance difference and serving as an indicator of prediction reliability. Colder hues signify high confidence levels, while warmer hues suggest lower confidence levels (Figure 2b).

**Figure 3:**
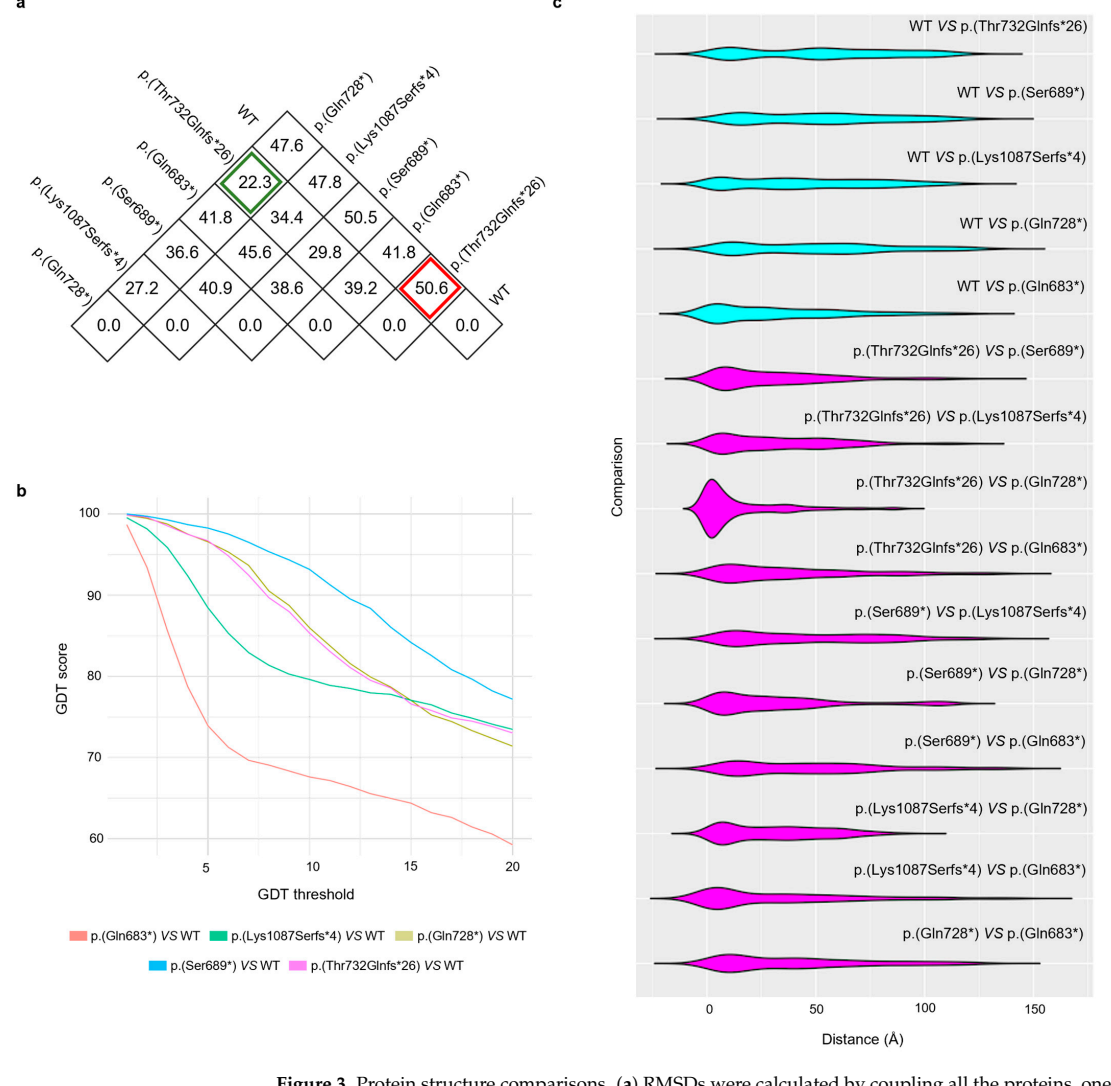
Protein structure comparisons. (**a**) RMSDs were calculated by coupling all the proteins, one versus another. Values are expressed in ångström. The red and green squares highlight the highest and the lowest values, respectively. (**b**) shows comparisons between the wild type and the proteins with ARHGAP31 variants using GDT. Values are expressed in ångström. (**c**) represents the violin plot with the distribution of distances for each compared couple. The first five violins are the comparisons of the wild type versus all the studied mutations. The other violins are the remaining comparisons. Values are expressed in ångström.

All tertiary structures were compared with each other using the RMSD (Figure 3a). The values in Figure 3a, expressed in ångström, are the root-mean-square distances for each comparison. The lowest value, 22.3 Å, can be observed for the comparison of p.(Gln728*) versus p.(Thr732Glnfs*26); on the contrary, p.(Thr732Glnfs*26) has the highest observable RMSD from the wild type, 50.6 Å (Figure 3a). In general, the RMSD values of the comparisons between the variants and the wild type range from 41.8 to 50.6 Å (Figure 3a). 

Moreover, we compared the ARHGAP31 variants and the wild type using the GDT (Figure 3b). Similarly, the trend of the global distance test shows an indicator that already differentiates for low values, always for the same comparison. As expected, the GDT decreases as the threshold used for distance acceptability increases (Figure 3b). At all thresholds, the GDT is comparable when we compare the wild-type structure with p.(Gln728*) or p.(Thr732Glnfs*26) (Figure 3b). In particular, the comparison between p.(Gln683*) and the wild type shows that the two proteins differ by 90% at a threshold value of ≤ 2.5 Å (Figure 3b). Meanwhile, the other variants, including p.(Thr732Glnfs*26), differ from the wild type by more than 95% at a threshold value of 2.5 Å (Figure 3b). It can be observed that at the minimum distance of 5 Å, the GDT drops to a ≤ 80% difference (Figure 3b). The comparison between the proteins and ARHGAP31 variants based on distance highlights that all the proteins are almost similar to each other, except for the protein that has a smaller difference in distance, as observed in Figure 3b. The comparison with the wild type shows that all the proteins with ARHGAP31 variants are comparable.

Similarly, in the violin plots (Figure 3c), it is possible to observe how the comparisons between each ARHGAP31 variant protein and the wild type show comparable trends, highlighting that the newly identified ARHGAP31 variant induces the production of a protein similar to other already investigated proteins.

### 3.4. Array CGH Analysis

**Figure 4:**
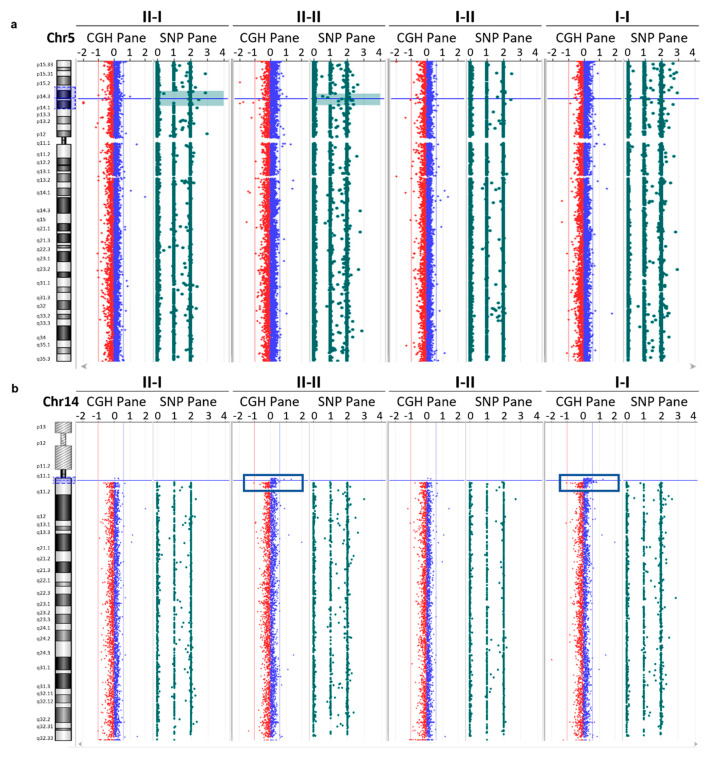
aCGH analysis. Panel (**a**) shows the array CGH analysis result in which the region of loss of heterozygosity (LOH) is highlighted at the level of the p14–p15 band of chromosome 5 in both siblings (II-I and II-II), with interstitial regions of 6 Mb and 9 Mb, respectively. Panel (**b**) shows the analysis result in which a DNA copy number gain of an interstitial region of about 1300 kb is identified at the level of the q11 band of chromosome 14 in the proband’s brother and father.

Since the clinical exome analysis was not able to identify CNVs, we completed our study performing an array CGH analysis using DNA derived from all the family members. Array CGH analysis showed a region of loss of heterozygosity (LOH) at the level of the p14–p15 band of chromosome 5 in both siblings (II-I and II-II), with interstitial regions of 6 Mb and 9 Mb, respectively (Figure 4a). Specifically, in the proband (II-I), this region of about 9197 kb covers the region between nucleotides 18055380 and 27253029. However, in the proband’s brother (II-II) this region of about 6907 kb covers the region between nucleotides 20021106 and 26920010. This LOH region in both siblings includes six OMIM genes: *CDH9*, *CDH10*, *CDH12*, *CDH18*, *PRDM9*, and *PMCHL1*. In addition, the genome-wide CNV analysis shows a genomic profile with a gain in the number of copies of DNA sequences in an interstitial region of about 1300 kb at the level of the q11 band of chromosome 14 in the son (II-II) inherited from the father (I-I) (Figure 4b). The duplicated region covers the region between nucleotides 19100682 and 20421677 and includes one OMIM gene: *POTEG*. This alteration is reported in the DGV, Franklin, and ClinVar databases as benign or a VUS. In accordance with the ACMG 2020 guidelines [10], we classified this variant as uncertain significance (Class 3), with a score of 0.90 (CNV interpretation scoring rubric).

### 3.5. Gene Expression Analysis

**Figure 5:**
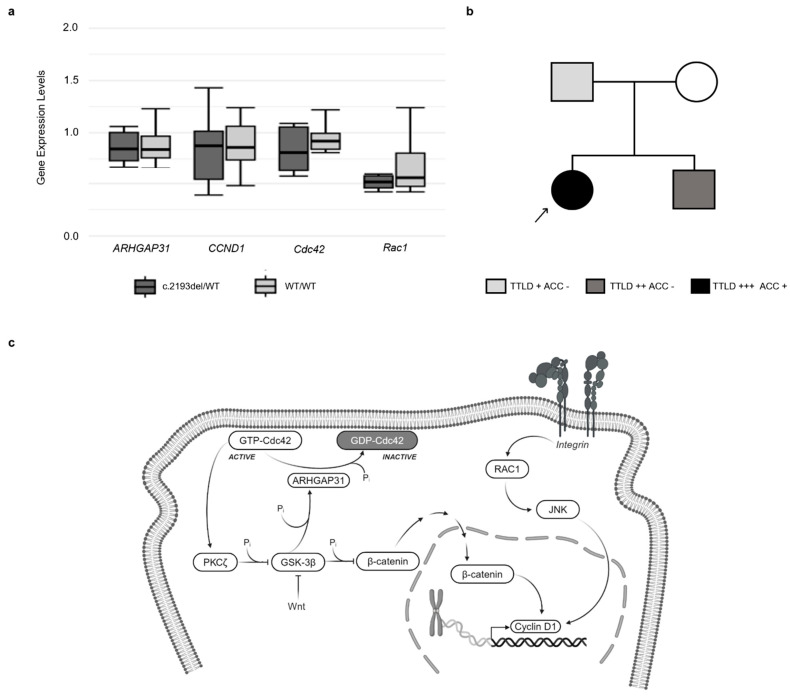
*ARHGAP31* variants and clinical variability in AOS patients. Gene expression analysis is shown in panel (**a**). No differences were identified in *ARHGAP31*, *CCND1*, *Cdc42*, and *Rac1* expression levels in patients and controls. (**b**) Family pedigree showing segregation of c.2193del p.(Thr732Glnfs*26) and the variability of the clinical spectrum characterizing the proband, her father, and her brother. Different colors indicate the variable phenotypic spectrum in the family. (**c**) Schematic representation of ARHGAP31 pathway. Created with BioRender.com.

To investigate *ARHGAP31* expression level alterations in patients with c.2193del, we performed *ARHGAP31* expression analysis in all family members and six control subjects without c.2193del. Moreover, we analyzed the mRNA levels of genes involved in the ARHGAP31 pathway, such as *CCND1*, *Cdc42*, and *Rac1*. No significant gene expression differences were identified between subjects with and without *ARHGAP31* variants (Figure 5a).

## 4. Discussion

In this study, we used a genome-wide and multidisciplinary approach to investigate the genetic defect underlying the foot abnormalities identified in a family with three subjects exhibiting a variable phenotype of lower limb defects (Figure 5b and Table 1). In the first clinical evaluation, the proband showed a severe terminal reduction defect in the feet, whereas her brother and father had a milder phenotype. In particular, the proband’s brother exhibited a shortening of the lower extreme digits of both feet, and the father had only a bilateral partial syndactyly of the second and third toes of both feet. When we performed a clinical exome sequencing analysis for all the members of this family, we found that the three patients with foot abnormalities showed a heterozygous variant in exon 12 of the *ARHGAP31* gene, c.2193del p.(Thr732Glnfs*26) (LOVD accession number: #0000972101). The identified alteration is a novel frameshift variant that leads to the premature termination of protein translation and the formation of a truncated protein with an altered C-terminal domain. 

Nonsense and frameshift variants in exon 12 of *ARHGAP31*, leading to a truncated C-terminal domain, have previously been reported and associated with dominant autosomal forms of AOS (Figure 2a). To our knowledge, missense variants of *ARHGAP31* that contribute to the development of dominant forms of AOS have not been described, yet. However, the synergic effect of two rare missense variants of the *ARHGAP31* and *FBLN1* genes was recently demonstrated in a patient with terminal transverse limb defects [19]. A heterozygous individual carrying one of these variants did not show the phenotype [19].

To our knowledge, deletions involving only the *ARGHAP31* gene have not been described in the literature or public databases. Some larger deletions are reported (UCSC Genome Browser) [20] to cause developmental delay and malformations, but they contain several additional genes, and the role of the sole *ARGHAP31* gene cannot be inferred. Caron et al. [21] demonstrated that the complete loss of *ARGHAP31* expression in mouse embryos leads to hypovascularization, vascular defects, and incomplete embryonic/perinatal lethality. 

Four pathogenic variants, with the same protein effect of the identified alteration (p.(Gln683*), p.(Ser689*), p.(Gln728*), and p.(Lys1087Serfs*4); Figure 2a), have been described in patients with limb abnormalities [3,6,7]. The protein inference helped us to understand the differences between the wild-type and mutated protein structures and the similarity between the known ARHGAP31 variants associated with AOS and the novel one, p.(Thr732Glnfs*26). It has been demonstrated that these types of variants do not induce the nonsense-mediated decay pathway, and they have a gain-of-function effect on the produced protein. ARHGAP31 is a RhoGAP protein involved in different cellular pathways, such as cell division and migration. Its C-terminal domain is essential for protein autoinhibition by a specific interaction with the N-terminal RhoGAP domain, hiding its catalytic site. A C-terminal-truncated protein lacks this mechanism, resulting in the exposure of its catalytic site and showing constitutive activation [6]. The constitutive activation of ARHGAP31 leads to the inhibition of the Rho-GTPase Cdc42 and Rac1, with the consequent impairment of different cellular processes (Figure 5c). Several studies have highlighted the significant role of these proteins in limb development: the deletion of Rac1 induced limb defects and syndactyly in Rac1 conditional knockout mice [22], and the limb bud mesenchyme-specific inactivation of Cdc42 induced short limbs and bodies, syndactyly, and abnormal cranium calcification in mice [23].

According to the ACMG and ACGS guidelines [8,9], the criteria that we can use to classify the new variant are PM2_Moderate, PM4_Moderate, and PP3_Supporting. PM2 was applied due to its absence in the control population (gnomAD). The ACGS guidelines suggest the use of PM2 as moderate evidence after an ACGS impact evaluation about the effect of PM2 downgrading [9]. PM4 was used because the variant is a truncating variant in the last exon of *ARHGAP31*, and it is predicted to cause a gain-of-function effect. PP3 was applied for in silico and bioinformatic analyses. The application of these criteria allowed us to classify this new variant as a variant of uncertain significance (VUS), with a score of five points. The ACGS guidelines propose a sub-classification of VUSs into three groups based on the score achieved. According to these categories, the new *ARHGAP31* variant could be considered a “hot” VUS (4–5 points).

Moreover, if we consider the data in the literature supporting the evidence of pathogenicity for truncating variants in the last exon of *ARHGAP31* [3,6] and the clinical features of the studied family, the newly identified variant is probably involved in AOS. The analysis of other family members and in vitro functional experiments could be decisive in clarifying the variant’s classification and its involvement in AOS.

In order to add information on the CNV, a CGH array analysis was performed on all the family members. This analysis showed, in both the proband and her brother with malformation defects in the feet, an LOH in the same interstitial region on chromosome 5 involving six OMIM genes. A correlation between this outcome and the clinical picture was suggested, given the extent of the LOH region and, especially, its presence in both siblings. However, the OMIM genes involved are not described in the literature as causative to any recessive disease that could justify the described phenotype. 

The detected duplication in the proband’s brother is also present in the father on chromosome 14 and involves only the OMIM gene: *POTEG*. This gene is not associated with any clinical picture described in the literature, and, therefore, it is not expected to be implicated in the clinical picture described in the subjects of the family studied.

The clinical exome analysis results suggested a clinical re-examination of the family members. The proband showed a mild thinning of the cutis at the palpation of the vertex region on her cranium. No other signs of AOS were found in the proband, her brother, or her father, highlighting the clinical variability in these patients. 

The wide phenotypic variability in patients with *ARHGAP31* variants has been described in other studies [7], highlighting syndrome manifestations ranging from severe and milder limb defects to an absence of clinical signs. Isrie et al. [7] classified AOS patients with *ARHGAP31* variants into three categories based on clinical variability: category A, patients with a severe phenotype; category B, patients with moderate–mild signs; and category C, clinically unaffected *ARHGAP31* variant carriers. These clinical categories can be also used for our patients. In particular, the proband could be assigned to category A, and her brother and father to B.

The clinical variability in these AOS patients suggests the reduced penetrance of this syndrome associated with *ARHGAP31* variants and points out the importance of *ARHGAP31* analysis in patients with terminal limb abnormalities, even if isolated. This is useful for genetic counseling for subjects asking for assessments of their reproductive risk, such as our proband.

## 5. Conclusions

In this study, we described a family with a novel truncating variant in *ARHGAP31* that is probably involved in an autosomal dominant form of AOS. We reported further evidence of the phenotypic variability of this syndrome in patients with variants in *ARHGAP31*. Further in vitro studies and protein analysis, such as Western blot experiments using patients’ PBMCs, and the analysis of other family members are needed to elucidate the molecular mechanisms of this new variant and its involvement in AOS’s variable clinical spectrum. However, we demonstrated that a multidisciplinary approach can be helpful for the characterization of variants involved in syndromes with a wide phenotypic spectrum like AOS.

## Figures and Tables

**Table 1:** Personal information and clinical records of the proband and her father and brother, to date. The table summarizes salient clinical signs identified in the family. NA: not-available data; +, ++, and +++: different severities in phenotypic manifestations; -: clinical symptom not present; AD: anomalies detected; AND: anomalies not detected.

	II-I	I-I	II-II
**Personal data**			
Date of birth	3 June 1989	31 August 1955	30 July 1985
Parenthood	Proband	Father	Brother
**Objective examination**			
Gender	F	M	M
Height (cm)	156	168	170
Foot abnormalities	+++	+	++
Hand abnormalities	+	-	-
Cranial alterations	+	-	-
**Instrumental examination**			
Foot’s radiography	AD	NA	NA
Electrocardiogram	AND	AND	AND
Sweat chloride test	AND	NA	NA

## Data Availability

The datasets supporting the current study have not been stored in a public repository, and anonymized data are available from the corresponding author upon reasonable request.

## Appendix A

AlphaFold, https://alphafold.ebi.ac.uk/ (accessed on 26 October 2023)
PDB, https://www.rcsb.org/ (accessed on 26 October 2023)
PDB70, https://wwwuser.gwdg.de/~compbiol/data/hhsuite/databases/hhsuite_dbs/ (accessed on 26 October 2023)
BFD, https://bfd.mmseqs.com/ (accessed on 26 October 2023)
MGnify, https://www.ebi.ac.uk/metagenomics (accessed on 26 October 2023)
Primer3Plus, https://primer3.org/ (accessed on 16 August 2022)
UniRef30 (FKA UniClust30), https://uniclust.mmseqs.com/ (accessed on 26 October 2023)
UniRef90, https://www.uniprot.org/help/uniref (accessed on 26 October 2023)
BioPython, http://www.biopython.org (accessed on 26 October 2023)
GnomAD, https://gnomad.broadinstitute.org/ (accessed on 5 April 2024)
Clinvar, https://www.ncbi.nlm.nih.gov/clinvar/ (accessed on 5 April 2024)
LOVD, https://www.lovd.nl/ (accessed on 5 April 2024)
